# Ethyl 5-acetyl-2-amino-4-methyl­thio­phene-3-carboxyl­ate

**DOI:** 10.1107/S1600536808014177

**Published:** 2008-05-17

**Authors:** Mehmet Akkurt, Sema Öztürk Yıldırım, Abdullah Mohamed Asiri, Vickie McKee

**Affiliations:** a Department of Physics, Faculty of Arts and Sciences, Erciyes University, 38039 Kayseri, Turkey; b Chemistry Department, Faculty of Science, King Abdul-Aziz University, PO Box 80203, Jeddah 21589, Saudi Arabia; c Department of Chemistry, Loughborough University, Leicestershire LE11 3TU, England

## Abstract

In the title compound, C_10_H_13_NO_3_S, prepared in a one-pot reaction, the mol­ecular conformation is stabilized by an intra­molecular N—H⋯O hydrogen bond. The packing is consolidated by further N—H⋯O links. The H atoms of two of the methyl groups are disordered over two sets of sites with equal occupancies.

## Related literature

For related literature, see: Gewald *et al.* (1966 ▶); Sabnis *et al.* (1999 ▶); Akkurt *et al.* (2008 ▶); Allen *et al.* (1987 ▶).
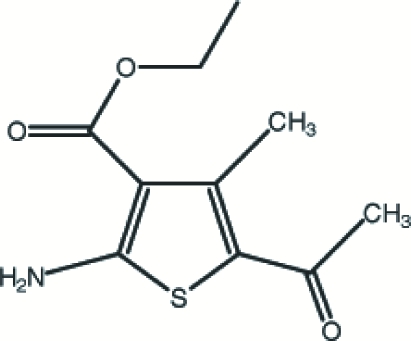


## Experimental

### 

#### Crystal data

C_10_H_13_NO_3_S
*M* *_r_* = 227.28
Monoclinic, 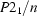

*a* = 7.5397 (3) Å
*b* = 8.4514 (3) Å
*c* = 16.7058 (6) Å
β = 94.465 (1)°
*V* = 1061.28 (7) Å^3^
*Z* = 4
Mo *K*α radiation
μ = 0.29 mm^−1^
*T* = 150 (2) K
0.29 × 0.26 × 0.10 mm

#### Data collection

Bruker APEXII CCD diffractometer
Absorption correction: multi-scan (*SADABS*; Sheldrick, 2003 ▶) *T*_min_ = 0.920, *T*_max_ = 0.971
12338 measured reflections
3400 independent reflections
2944 reflections with *I* > 2σ(*I*)
*R*_int_ = 0.025

#### Refinement

*R*[*F* ^2^ > 2σ(*F* ^2^)] = 0.038
*wR*(*F* ^2^) = 0.111
*S* = 1.05
3400 reflections
136 parameters
H-atom parameters constrained
Δρ_max_ = 0.44 e Å^−3^
Δρ_min_ = −0.34 e Å^−3^

### 

Data collection: *APEX2* (Bruker, 2005 ▶); cell refinement: *APEX2*; data reduction: *APEX2*; program(s) used to solve structure: *SIR97* (Altomare *et al.*, 1999 ▶); program(s) used to refine structure: *SHELXL97* (Sheldrick, 2008 ▶); molecular graphics: *ORTEP-3 for Windows* (Farrugia, 1997 ▶); software used to prepare material for publication: *WinGX* (Farrugia, 1999 ▶).

## Supplementary Material

Crystal structure: contains datablocks global, I. DOI: 10.1107/S1600536808014177/hb2733sup1.cif

Structure factors: contains datablocks I. DOI: 10.1107/S1600536808014177/hb2733Isup2.hkl

Additional supplementary materials:  crystallographic information; 3D view; checkCIF report

## Figures and Tables

**Table 1:** Hydrogen-bond geometry (Å, °)

*D*—H⋯*A*	*D*—H	H⋯*A*	*D*⋯*A*	*D*—H⋯*A*
N1—H*N*1*A*⋯O2	0.86	2.15	2.7404 (14)	125
N1—H*N*1*A*⋯O2^i^	0.86	2.40	3.2077 (15)	156
N1—H*N*1*B*⋯O1^ii^	0.86	2.24	2.9933 (14)	147
C5—H5*A*⋯O3	0.96	2.04	2.7978 (16)	135

Symmetry codes: (i) 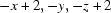; (ii) 
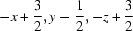
.

**Fig. 1.**
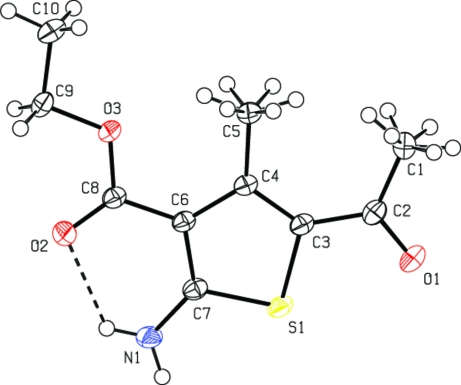
View of the molecular structure of (I) with displacement ellipsoids for non-H atoms drawn at the 50% probability level. The hydrogen bond is shown as a dashed line.

**Fig. 2.**
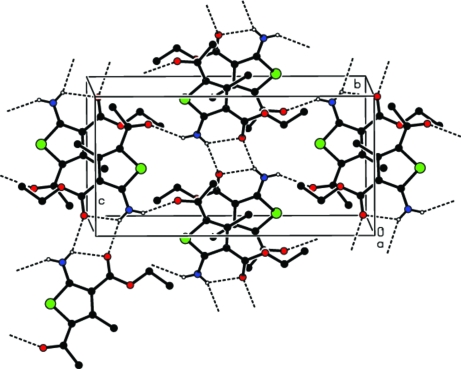
View of the packing and hydrogen bonding in (I).

## supplementary crystallographic information

### Comment

2-Aminothiophene derivatives are important intermediates in the synthesis of a variety of agrochemicals, dyes and pharmacologically active compounds (Sabnis *et al.*, 1999). The most convergent and well established classical approach for the preparation of 2-aminothiophenes is Gewald's method (Gewald *et al.*, 1966), which involves the multicomponent condensation of a ketone with an activated nitrile and elemental sulfur in the presence of diethylamine as a catalyst.

As a part of an ongoing investigation into the development of anil derivatives, we here report the structure of the title compound, (I).

All bond lengths and angles in (I) (Fig. 1) are within their normal ranges (Akkurt *et al.*, 2008; Allen *et al.*, 1987). The thiophene ring is almost planar, with a maximum deviation of -0.009 (1) Å for C6. The structure is stabilized by weak intra molecular C—H···O and N—H···O, and intermolecular N—H···O hydrogen bonding interactions (Table 1 and Fig. 2).

### Experimental

A mixture of ethyl cyanoacetate (11.3 g, 0.10 mol) and acetyl acetone (10.22 g, 0.10 mol) in absolute ethanol (20 ml) was added to a solution of elemental sulfur (3.2 g, 0.10 mol) and diethylamine (5 ml) in 50 ml absolute ethanol at room temperature. The reaction mixture was refluxed for 3 h and then cooled. The precipitated product was filtered, washed with ethanol, dried and recrystallized from ethanol as orange blocks of (I) [yield: 52%, m.p. 435-437 K]. IR (cm^-1^) 3408, 3294 (NH), 1666 (CO), 1605, 1586,1253. ^1^H-NMR (CDCl_3_): 1.38 (t, 3H, CH3CH_2_O),2.43 (s, 3H, COCH_3_), 2.7 (s, 3H, CH_3_), 4.32 (q, 2H, OCH_2_),6.67 (broad s, 2H, NH_2_).

### Refinement

All the H atoms were positioned geometrically (C—H = 0.96 - 0.97 Å and N—H = 0.86 Å) and refined as riding with *U*_iso_ = 1.2*U*_eq_(carrier) (1.5*U*_eq_ for methyl C). The methyl H atoms attached to C1 and C5 were refined as disordered over two sets of sites.

### Crystal data

**Table:**

C_10_H_13_NO_3_S	*F*(000) = 480
*M**_r_* = 227.28	*D*_x_ = 1.423 Mg m^−^^3^
Monoclinic, *P*2_1_/*n*	Mo *K*α radiation, λ = 0.71073 Å
Hall symbol: -P 2yn	Cell parameters from 4913 reflections
*a* = 7.5397 (3) Å	θ = 2.5–31.1°
*b* = 8.4514 (3) Å	µ = 0.29 mm^−^^1^
*c* = 16.7058 (6) Å	*T* = 150 K
β = 94.465 (1)°	Block, orange
*V* = 1061.28 (7) Å^3^	0.29 × 0.26 × 0.10 mm
*Z* = 4	

### Data collection

**Table:**

Bruker APEXII CCD diffractometer	3400 independent reflections
Radiation source: sealed tube	2944 reflections with *I* > 2σ(*I*)
graphite	*R*_int_ = 0.025
φ and ω scans	θ_max_ = 31.8°, θ_min_ = 2.5°
Absorption correction: multi-scan (*SADABS*; Sheldrick, 2003)	*h* = −11→10
*T*_min_ = 0.920, *T*_max_ = 0.971	*k* = −12→12
12338 measured reflections	*l* = −23→24

### Refinement

**Table:**

Refinement on *F*^2^	Primary atom site location: structure-invariant direct methods
Least-squares matrix: full	Secondary atom site location: difference Fourier map
*R*[*F*^2^ > 2σ(*F*^2^)] = 0.038	Hydrogen site location: inferred from neighbouring sites
*wR*(*F*^2^) = 0.111	H-atom parameters constrained
*S* = 1.05	*w* = 1/[σ^2^(*F*_o_^2^) + (0.06*P*)^2^ + 0.3751*P*] where *P* = (*F*_o_^2^ + 2*F*_c_^2^)/3
3400 reflections	(Δ/σ)_max_ = 0.001
136 parameters	Δρ_max_ = 0.44 e Å^−^^3^
0 restraints	Δρ_min_ = −0.34 e Å^−^^3^

### Special details

**Table:**

Geometry. Bond distances, angles *etc*. have been calculated using the rounded fractional coordinates. All su's are estimated from the variances of the (full) variance-covariance matrix. The cell e.s.d.'s are taken into account in the estimation of distances, angles and torsion angles
Refinement. Refinement on *F*^2^ for ALL reflections except those flagged by the user for potential systematic errors. Weighted *R*-factors *wR* and all goodnesses of fit *S* are based on *F*^2^, conventional *R*-factors *R* are based on *F*, with *F* set to zero for negative *F*^2^. The observed criterion of *F*^2^ > σ(*F*^2^) is used only for calculating -*R*-factor-obs *etc*. and is not relevant to the choice of reflections for refinement. *R*-factors based on *F*^2^ are statistically about twice as large as those based on *F*, and *R*-factors based on ALL data will be even larger.

### Fractional atomic coordinates and isotropic or equivalent isotropic displacement parameters (Å^2^)

**Table:**

	*x*	*y*	*z*	*U*_iso_*/*U*_eq_	Occ. (<1)
S1	0.74240 (4)	0.44716 (4)	0.82882 (2)	0.0234 (1)	
O1	0.58575 (14)	0.74136 (13)	0.79734 (6)	0.0324 (3)	
O2	0.94110 (15)	0.13922 (12)	1.03979 (6)	0.0315 (3)	
O3	0.83808 (13)	0.32842 (11)	1.11776 (5)	0.0251 (3)	
N1	0.88307 (16)	0.17090 (13)	0.87660 (6)	0.0263 (3)	
C1	0.5682 (2)	0.86338 (17)	0.92406 (8)	0.0307 (4)	
C2	0.61541 (17)	0.72854 (15)	0.87090 (7)	0.0240 (3)	
C3	0.69568 (16)	0.58332 (15)	0.90307 (7)	0.0210 (3)	
C4	0.74019 (15)	0.52653 (14)	0.97964 (7)	0.0191 (3)	
C5	0.71928 (18)	0.61996 (15)	1.05495 (7)	0.0249 (3)	
C6	0.81050 (15)	0.36847 (14)	0.97838 (7)	0.0194 (3)	
C7	0.82188 (16)	0.31123 (14)	0.89967 (7)	0.0208 (3)	
C8	0.87045 (16)	0.26784 (14)	1.04635 (7)	0.0207 (3)	
C9	0.90152 (19)	0.23865 (16)	1.18821 (7)	0.0272 (3)	
C10	0.8610 (2)	0.33700 (19)	1.25956 (8)	0.0325 (4)	
HN1A	0.92190	0.10290	0.91200	0.0320*	
H1A	0.51730	0.94800	0.89160	0.0460*	0.500
H1B	0.67360	0.90040	0.95430	0.0460*	0.500
H1C	0.48370	0.82780	0.96020	0.0460*	0.500
H1D	0.59910	0.83610	0.97920	0.0460*	0.500
H1E	0.44280	0.88380	0.91640	0.0460*	0.500
H1F	0.63260	0.95630	0.91050	0.0460*	0.500
HN1B	0.88350	0.14890	0.82640	0.0320*	
H5A	0.75800	0.55710	1.10090	0.0370*	0.500
H5B	0.59650	0.64800	1.05770	0.0370*	0.500
H5C	0.78990	0.71440	1.05430	0.0370*	0.500
H5D	0.67160	0.72250	1.04100	0.0370*	0.500
H5E	0.83320	0.63170	1.08430	0.0370*	0.500
H5F	0.63970	0.56530	1.08760	0.0370*	0.500
H9A	1.02850	0.21990	1.18830	0.0330*	
H9B	0.84120	0.13740	1.18920	0.0330*	
H10A	0.90050	0.28220	1.30800	0.0490*	
H10B	0.73510	0.35490	1.25850	0.0490*	
H10C	0.92150	0.43670	1.25770	0.0490*	

### Atomic displacement parameters (Å^2^)

**Table:**

	*U*^11^	*U*^22^	*U*^33^	*U*^12^	*U*^13^	*U*^23^
S1	0.0306 (2)	0.0254 (2)	0.0142 (1)	−0.0032 (1)	0.0017 (1)	−0.0009 (1)
O1	0.0409 (6)	0.0366 (5)	0.0198 (4)	0.0027 (4)	0.0031 (4)	0.0076 (4)
O2	0.0468 (6)	0.0238 (4)	0.0241 (5)	0.0083 (4)	0.0037 (4)	−0.0003 (3)
O3	0.0348 (5)	0.0258 (4)	0.0147 (4)	0.0055 (4)	0.0015 (3)	0.0012 (3)
N1	0.0373 (6)	0.0222 (5)	0.0197 (5)	−0.0009 (4)	0.0038 (4)	−0.0052 (4)
C1	0.0386 (7)	0.0263 (6)	0.0271 (6)	0.0064 (5)	0.0016 (5)	0.0038 (5)
C2	0.0248 (5)	0.0262 (6)	0.0212 (5)	−0.0031 (4)	0.0027 (4)	0.0040 (4)
C3	0.0245 (5)	0.0224 (5)	0.0163 (5)	−0.0032 (4)	0.0023 (4)	−0.0006 (4)
C4	0.0200 (5)	0.0211 (5)	0.0164 (5)	−0.0030 (4)	0.0022 (4)	−0.0010 (4)
C5	0.0327 (6)	0.0246 (6)	0.0174 (5)	0.0024 (5)	0.0017 (4)	−0.0026 (4)
C6	0.0216 (5)	0.0206 (5)	0.0161 (5)	−0.0026 (4)	0.0018 (4)	−0.0013 (4)
C7	0.0228 (5)	0.0217 (5)	0.0180 (5)	−0.0045 (4)	0.0024 (4)	−0.0017 (4)
C8	0.0234 (5)	0.0215 (5)	0.0173 (5)	−0.0025 (4)	0.0019 (4)	−0.0008 (4)
C9	0.0360 (7)	0.0270 (6)	0.0185 (5)	0.0027 (5)	0.0010 (5)	0.0057 (4)
C10	0.0407 (7)	0.0392 (7)	0.0179 (5)	0.0024 (6)	0.0044 (5)	0.0023 (5)

### Geometric parameters (Å, °)

**Table:**

S1—C3	1.7479 (13)	C1—H1A	0.9600
S1—C7	1.7232 (12)	C1—H1B	0.9600
O1—C2	1.2366 (15)	C1—H1C	0.9600
O2—C8	1.2192 (16)	C1—H1D	0.9600
O3—C8	1.3380 (15)	C1—H1E	0.9600
O3—C9	1.4498 (15)	C1—H1F	0.9600
N1—C7	1.3400 (16)	C5—H5A	0.9600
N1—HN1A	0.8600	C5—H5B	0.9600
N1—HN1B	0.8600	C5—H5C	0.9600
C1—C2	1.5044 (19)	C5—H5D	0.9600
C2—C3	1.4529 (18)	C5—H5E	0.9600
C3—C4	1.3829 (17)	C5—H5F	0.9600
C4—C6	1.4379 (17)	C9—H9A	0.9700
C4—C5	1.5040 (17)	C9—H9B	0.9700
C6—C7	1.4100 (17)	C10—H10A	0.9600
C6—C8	1.4619 (17)	C10—H10B	0.9600
C9—C10	1.5039 (19)	C10—H10C	0.9600
			
C3—S1—C7	91.72 (6)	H1C—C1—H1D	56.00
C8—O3—C9	116.87 (10)	H1C—C1—H1E	56.00
HN1A—N1—HN1B	120.00	H1C—C1—H1F	141.00
C7—N1—HN1A	120.00	H1D—C1—H1E	110.00
C7—N1—HN1B	120.00	H1D—C1—H1F	109.00
O1—C2—C1	119.18 (12)	H1E—C1—H1F	109.00
O1—C2—C3	118.66 (11)	C4—C5—H5A	109.00
C1—C2—C3	122.17 (11)	C4—C5—H5B	110.00
S1—C3—C2	113.26 (9)	C4—C5—H5C	109.00
C2—C3—C4	134.38 (11)	C4—C5—H5D	109.00
S1—C3—C4	112.34 (9)	C4—C5—H5E	109.00
C5—C4—C6	124.25 (10)	C4—C5—H5F	109.00
C3—C4—C6	111.84 (10)	H5A—C5—H5B	109.00
C3—C4—C5	123.91 (11)	H5A—C5—H5C	110.00
C4—C6—C7	112.44 (10)	H5A—C5—H5D	141.00
C4—C6—C8	128.40 (11)	H5A—C5—H5E	56.00
C7—C6—C8	119.16 (10)	H5A—C5—H5F	56.00
N1—C7—C6	128.26 (11)	H5B—C5—H5C	109.00
S1—C7—N1	120.11 (9)	H5B—C5—H5D	56.00
S1—C7—C6	111.64 (9)	H5B—C5—H5E	141.00
O2—C8—O3	122.18 (11)	H5B—C5—H5F	56.00
O2—C8—C6	124.04 (11)	H5C—C5—H5D	56.00
O3—C8—C6	113.77 (10)	H5C—C5—H5E	56.00
O3—C9—C10	106.24 (11)	H5C—C5—H5F	141.00
C2—C1—H1A	109.00	H5D—C5—H5E	109.00
C2—C1—H1B	109.00	H5D—C5—H5F	109.00
C2—C1—H1C	109.00	H5E—C5—H5F	109.00
C2—C1—H1D	109.00	O3—C9—H9A	110.00
C2—C1—H1E	109.00	O3—C9—H9B	110.00
C2—C1—H1F	109.00	C10—C9—H9A	111.00
H1A—C1—H1B	109.00	C10—C9—H9B	110.00
H1A—C1—H1C	109.00	H9A—C9—H9B	109.00
H1A—C1—H1D	141.00	C9—C10—H10A	109.00
H1A—C1—H1E	56.00	C9—C10—H10B	110.00
H1A—C1—H1F	56.00	C9—C10—H10C	109.00
H1B—C1—H1C	110.00	H10A—C10—H10B	109.00
H1B—C1—H1D	56.00	H10A—C10—H10C	110.00
H1B—C1—H1E	141.00	H10B—C10—H10C	109.00
H1B—C1—H1F	56.00		
			
C7—S1—C3—C2	−178.44 (10)	C2—C3—C4—C6	177.16 (13)
C7—S1—C3—C4	0.21 (10)	C3—C4—C6—C7	1.69 (15)
C3—S1—C7—N1	−179.68 (11)	C3—C4—C6—C8	−179.30 (12)
C3—S1—C7—C6	0.76 (10)	C5—C4—C6—C7	−177.67 (11)
C9—O3—C8—O2	3.93 (18)	C5—C4—C6—C8	1.35 (19)
C9—O3—C8—C6	−177.14 (10)	C4—C6—C7—S1	−1.52 (13)
C8—O3—C9—C10	175.85 (11)	C4—C6—C7—N1	178.96 (12)
O1—C2—C3—S1	1.18 (16)	C8—C6—C7—S1	179.36 (9)
O1—C2—C3—C4	−177.07 (13)	C8—C6—C7—N1	−0.2 (2)
C1—C2—C3—S1	−178.56 (10)	C4—C6—C8—O2	−174.17 (12)
C1—C2—C3—C4	3.2 (2)	C4—C6—C8—O3	6.93 (18)
S1—C3—C4—C5	178.25 (10)	C7—C6—C8—O2	4.79 (19)
S1—C3—C4—C6	−1.10 (13)	C7—C6—C8—O3	−174.12 (11)
C2—C3—C4—C5	−3.5 (2)		

### Hydrogen-bond geometry (Å, °)

**Table:**

*D*—H···*A*	*D*—H	H···*A*	*D*···*A*	*D*—H···*A*
N1—HN1A···O2	0.86	2.15	2.7404 (14)	125
N1—HN1A···O2^i^	0.86	2.40	3.2077 (15)	156
N1—HN1B···O1^ii^	0.86	2.24	2.9933 (14)	147
C5—H5A···O3	0.96	2.04	2.7978 (16)	135

Symmetry codes: (i) −*x*+2, −*y*, −*z*+2; (ii) −*x*+3/2, *y*−1/2, −*z*+3/2.
